# Thyroid Nodule Classification in Ultrasound Images by Fine-Tuning Deep Convolutional Neural Network

**DOI:** 10.1007/s10278-017-9997-y

**Published:** 2017-07-10

**Authors:** Jianning Chi, Ekta Walia, Paul Babyn, Jimmy Wang, Gary Groot, Mark Eramian

**Affiliations:** 1 Department of Computer Science, University of Saskatchewan, 176 Thorvaldson Bldg, 110 Science Place, Saskatoon, SK S7N 5C9 Canada; 2 Department of Medical Imaging, University of Saskatchewan, 103 Hospital Dr, Saskatoon, SK S7N 0W8 Canada; 3 Department of Surgery, Royal University Hospital, 103 Hospital Drive, Suite 2646, Saskatoon, SK S7N 0W8 Canada

**Keywords:** Ultrasonography, Thyroid nodules, Machine learning, Computer vision, Deep learning, Convolutional neural network, Fine-tuning

## Abstract

With many thyroid nodules being incidentally detected, it is important to identify as many malignant nodules as possible while excluding those that are highly likely to be benign from fine needle aspiration (FNA) biopsies or surgeries. This paper presents a computer-aided diagnosis (CAD) system for classifying thyroid nodules in ultrasound images. We use deep learning approach to extract features from thyroid ultrasound images. Ultrasound images are pre-processed to calibrate their scale and remove the artifacts. A pre-trained GoogLeNet model is then fine-tuned using the pre-processed image samples which leads to superior feature extraction. The extracted features of the thyroid ultrasound images are sent to a Cost-sensitive Random Forest classifier to classify the images into “malignant” and “benign” cases. The experimental results show the proposed fine-tuned GoogLeNet model achieves excellent classification performance, attaining 98.29% classification accuracy, 99.10% sensitivity and 93.90% specificity for the images in an open access database (Pedraza et al. 16), while 96.34% classification accuracy, 86% sensitivity and 99% specificity for the images in our local health region database.

## Background

Detection of thyroid nodules (TNs) has significantly increased over the past two decades with many more nodules now being incidentally detected. As the majority of these nodules are benign or behave indolently, accurate determination of whether thyroid nodules are benign or malignant could reduce patient risk and reduce the significant medical health care costs with fine needle analysis (FNA) biopsy and/or surgery. The workup of these incidental thyroid nodules typically includes sonography. Radiologists have identified a few sonographic characteristics of thyroid nodules as suggestive features of malignancy, including hypo-echogenicity, absence of a halo, micro-calcifications, solidity, intra-nodular flow and taller-than-wide shape [1]. Based on these characteristics, a dedicated Thyroid Imaging Reporting and Data System (TI-RADS) [2] to categorize thyroid nodules and stratify their malignancy risk has been developed for used by radiologists. TI-RADS scores of 2, 3, 4a, 4b, 4c and 5 are denoted as “not suspicious”, “probably benign”, “one suspicious feature”, “two suspicious features”, “three or more suspicious features” and “probable malignancy”, respectively. However, the assessment of TNs using TI-RADS is time consuming and often not robust. The accuracy is often based on radiologists’ personal experience, as current sonographic criteria to identify malignant nodules are imperfect with the variations in echo patterns of thyroid nodules limiting the judgement capability of radiologists [3].

Introduction of a TI-RADS reporting scheme that is validated would allow clinicians to be able to stop regular thyroid ultrasound evaluation with confidence that they are unlikely to be missing a malignancy. It would also provide clinicians with a recommended follow-up plan with an intermediate risk TI-RADS score, saving the system a significant number of unnecessary imaging tests and the patients the anxiety of the associated uncertainty. Moreover, utilizing TI-RADS scores allows the radiologists to be aware of how the machine is classifying the image because the sonographic characteristics are contained in the ultrasound images that can be digitized and sent to machine learning scheme. Therefore, an automatic or semi-automatic classification system based on image features would be possible and very helpful to report TI-RADS scores and classify the thyroid nodule ultrasound images.

**Fig. 1:**
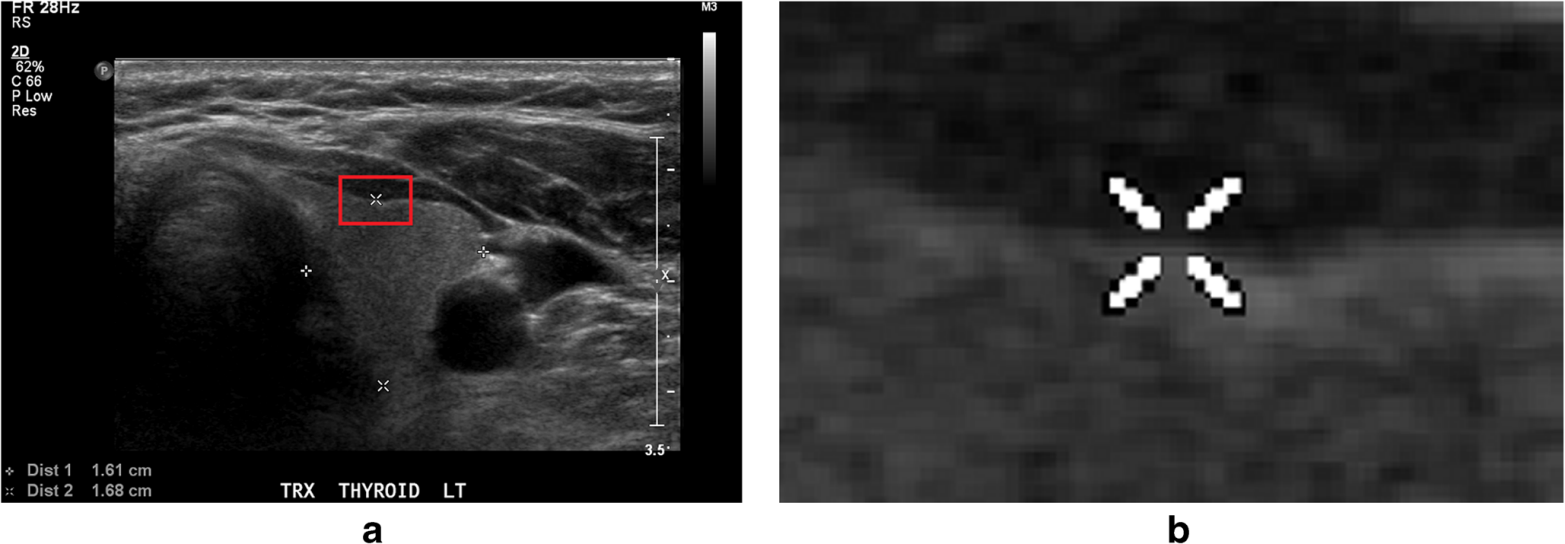
Example of artifacts made by radiologist on the thyroid ultrasound image. **a** Ultrasound image with artifacts covering the textures. **b** Details of how the artifact covers the ultrasound textures in the image, bounded by the *red rectangle* in **a**

Many works utilizing different hand-crafted features extracted from thyroid ultrasound images have been recently proposed [4–9]. These include the use of extracted features to perform supervised classification through existing machine learning classifiers. Most works used texture-based features in combination with a Support Vector Machine (SVM) classifier in order to accomplish the tasks of identification of nodules, classification of nodules based on their malignancy risk and characterization of the type of malignancy in a nodule [10]. The advantage and importance of computer-determined image features have been established in a recent study [11] where the authors concluded that non-clinical features such as tissue stiffness scores, texture and discrete-wavelet-transform-based features resulted in significantly higher classification accuracies compared to those obtained by clinical features alone such as vascularity, margins, shape, micro-calcifications, etc. Recently, image features extracted from deep convolutional neural network (DCNN) have proven effective in image classification, segmentation or retrieval. Compared to the traditional feature extraction methods, it was claimed in [12] that DCNN had two advantages: (1) detection using DCNN is robust to distortions such as changes in shape due to camera lens, different lighting conditions, different poses, presence of partial occlusions, horizontal and vertical shifts, etc.; (2) the computational cost of feature extraction by DCNN is relatively low because the same coefficients in the convolutional layer are used across the input image. Motivated by their observed advantages for non-medical images, DCNNs were applied to several medical image classification and detection problems. For example, Spanhol et al. [13] proposed the use of DCNN to classify the breast cancer histopathological images, Li et al. [14] proposed a pulmonary nodule classification system based on DCNN, and Roth et al. [15] proposed the use of DCNN to develop a lymph node detection system. Based on the successes of DCNNs on medical images seen in the literature, we sought to apply DCNN to classify thyroid ultrasound images. However, the images (discussed in more details below) used to train our specific DCNN model presented some problems: (1) our images were collected from different medical institutions so the texture scales varied across the data set, and (2) as shown in Fig. 1, artifacts such as fiducial markers added by radiologists degraded the learning process. Therefore, some pre-processing methods to enhance the image qualities and suitable augmentation of image samples would improve the performance of fine-tuned DCNN in classifying thyroid ultrasound images.

## Objective

Our objective was to develop a complete thyroid ultrasound image classification system based on a fine-tuned GoogLeNet model. The process of doing so included generating a more balanced thyroid nodule ultrasound image database, pre-processing the images for better image quality and fine-tuning GoogLeNet with the augmented image samples.

## Methods

### Image Collection

The images used in our research work were from the following two databases:

- Database 1 is a publicly available thyroid ultrasound image database proposed by Pedraza et al. [16], consisting of 428 thyroid ultrasound images with the size 560 × 360, of which 357 cases are labelled as positive (with the TI-RADS scores 3, 4a, 4b or 5), while 71 cases are labelled as negative (with the TI-RADS scores 1 or 2). The images were extracted from thyroid ultrasound video sequences captured with a TOSHIBA Nemio 30 and a TOSHIBA Nemio MX Ultrasound devices, both set to 12 MHz convex and linear transductors, containing the most relevant pathological features and their pathologies confirmed by biopsy using the BETHESDA system. The experts independently evaluated the patient individually and described the specific features filling the TI-RADS requirements;
- Database 2 is a local database, consisting of 164 thyroid ultrasound images, of which 122 images are with the sizes 1024(±5) × 695(±5), while 42 images are with the sizes 640(±5) × 440(±5). Thirty-five cases of the images in the database are labelled as positive (with TI-RADS scores of 3, 4a, 4b or 5), while 129 cases are labelled as negative (with TI-RADS scores of 1 or 2). The TI-RADS scores were evaluated by the local experts using the same method as those in database 1.

**Fig. 2:**
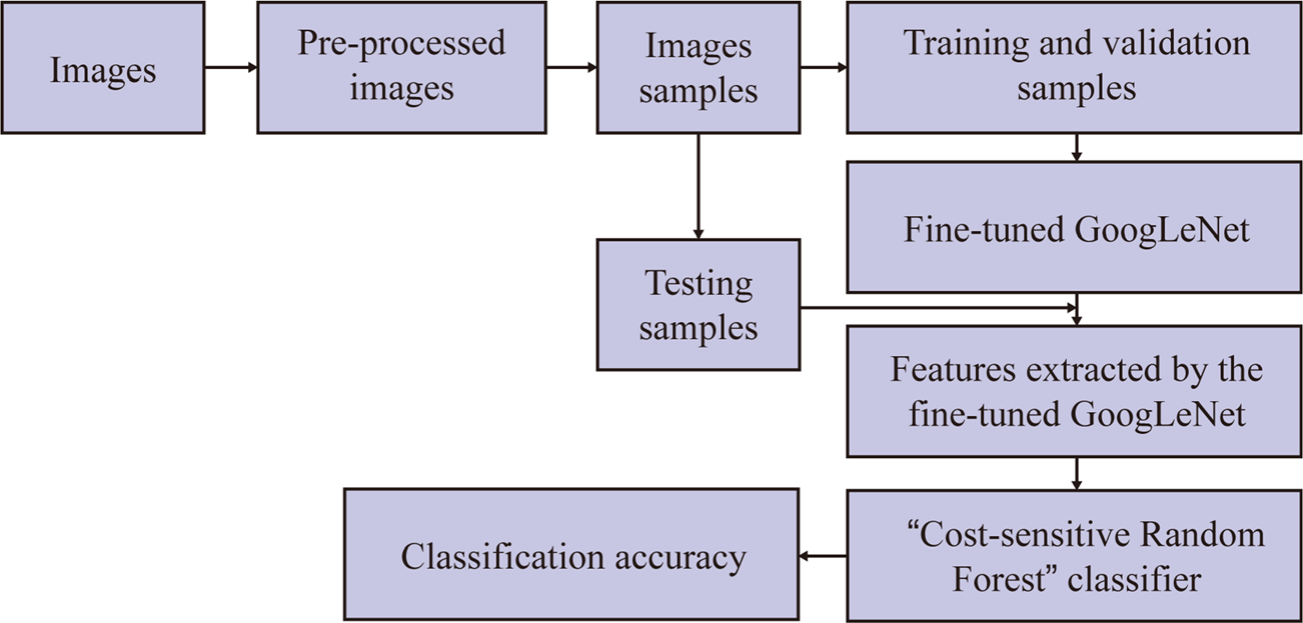
The process of the thyroid images classification based on fine-tuned GoogLeNet network

All the images from the two databases are 8-bit and 3-channel images, so they can be used as the training and validating images to fine-tune the DCNN directly. However, necessary image pre-processing algorithms were implemented to enhance the image qualities for better deep learning results. Figure 2 shows the process of the whole thyroid ultrasound image classification system.

### Image Pre-processing

**Fig. 3:**
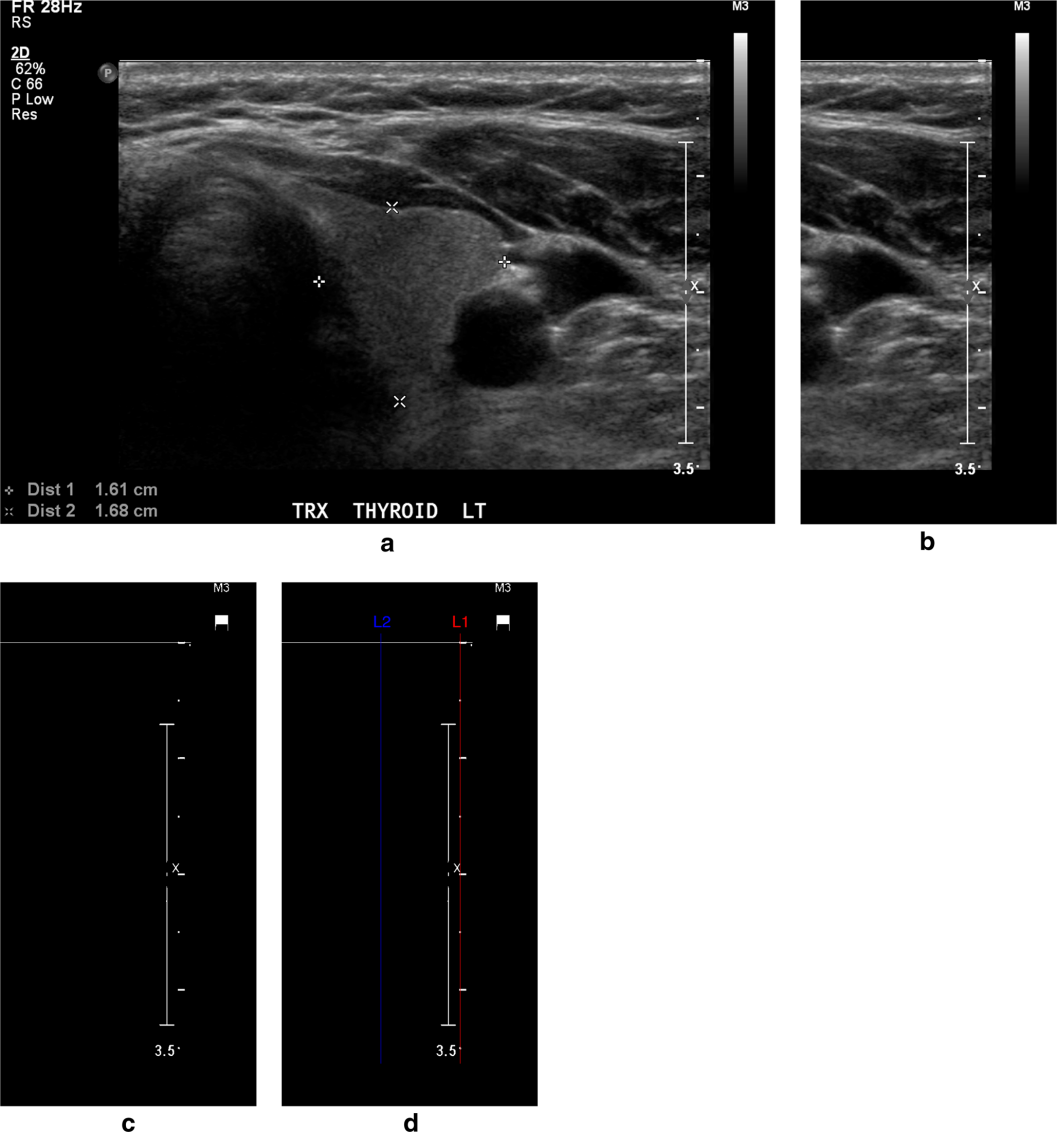
Detection of pixels deemed to be ticks by intensity thresholding. **a** Input thyroid ultrasound image. **b** Thirty percent right region of the input image where the thresholding was applied. **c** Binary image showing the pixels deemed likely to be ticks, and *red line* in **d** represents the column containing the tick bar

The images were firstly re-sized to have the same distance scale which is the physical space represented by each pixel in the image. The algorithm from [17] was implemented to find the column containing the tick bars in the ultrasound image and the tick spacing. As shown in Fig. 3, predicted ticks were found using the adaptive thresholding method, then the column containing “true” ticks and the corresponding tick distance *m* were identified based on the autocorrelation coefficients of each column of the predicted ticks. After we obtained all the gradation distances of the images in the database, we re-sized all the images using the image re-sizing function “imresize()” in MATLAB2014b, where the re-scaling coefficient for each image was the ratio of the largest tick distance in the database to the current tick distance, and the pixel intensity interpolating method was the “bi-cubic” interpolation scheme.

**Fig. 4:**
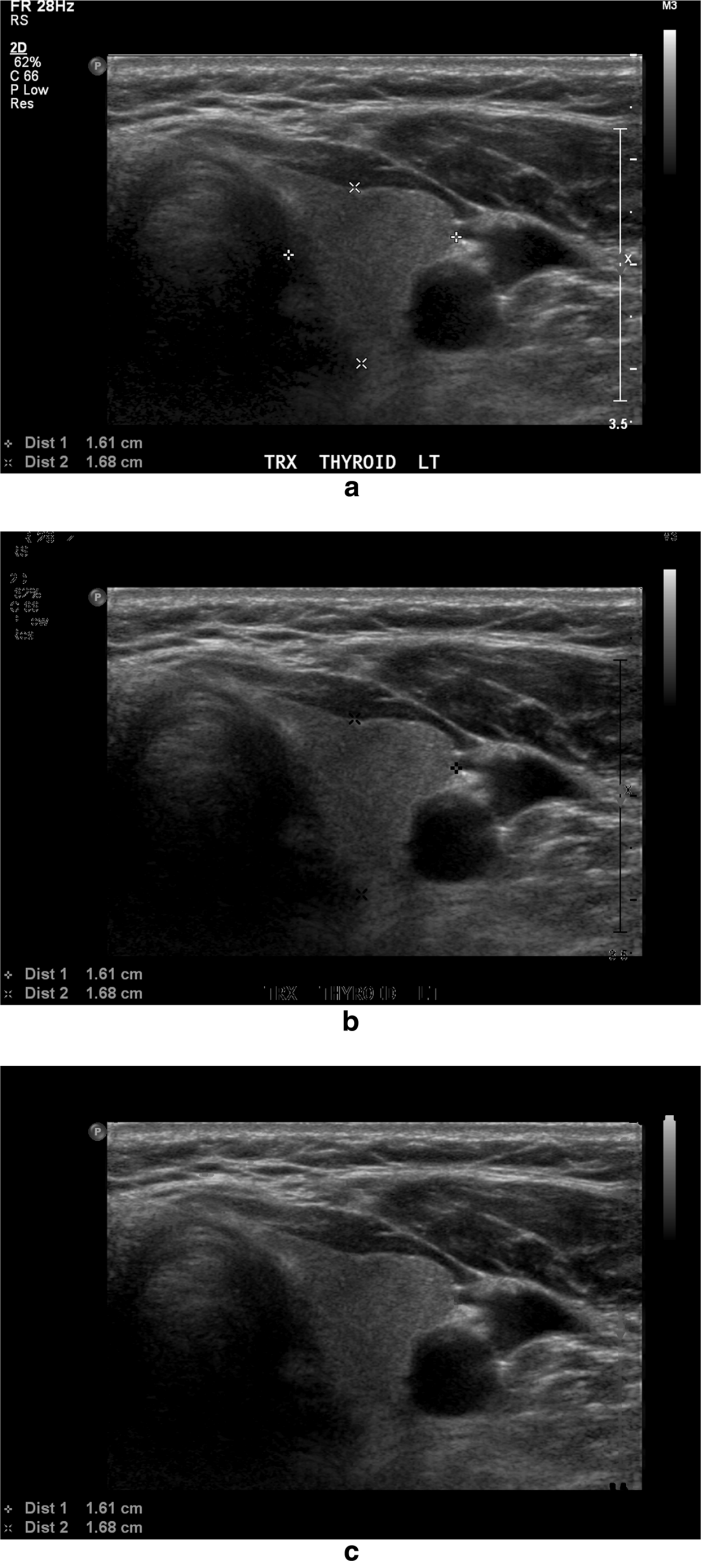
Example of removing artifacts from the thyroid image. **a** Original thyroid image with artifacts on the textures. **b** Thyroid image after removing the artifacts by the thresholding method. **c** Thyroid image after restoring the artifact-removed regions with textures similar to neighbourhood

The second image pre-processing phase was to remove annotations, such as the calliper markers drawn to indicate nodule size and region of interest. In our work, we followed the algorithm proposed by Narayan et al. [18] to remove the artifacts overlapping the region of interest and restore the gaps with the textures approximated from the surrounding regions. For a given input ultrasound image, the artifacts were removed as follows: (1) extracted all the non-zero regions as a binary image from the input image, (2) applied 2D connected component algorithm on the binary image, resulting in a labelled image with *K* components, (3) identified the texture region as the largest 2D connected component in the labelled image and subtracted it from the image to get the “possible artifact” regions, (4) plotted the histogram of the “possible artifact” regions and divided the histogram into three parts [0,100], [101,200], [201,255], respectively, (5) took the histogram peaks in each of the three parts as the intensity levels of the artifacts and (6) detected the artifacts as the pixels with those intensity levels and subtracted from the image to generate the artifact-removed image. The image with artifacts removed was then restored by implementing the method of Projection onto Convex Sets (POCS) [19] in MATLAB2014b. The algorithm retained the texture regions of the image and replaced the formerly artifact regions by the Block Discrete Cosine Transform of the neighbouring regions. Figure 4 shows the final output image after pre-processing steps.

### Sample Augmentation

Database 1 includes 428 images in total while database 2 includes 164 images in total, which are not enough from the requirement of image quantity for fine-tuning an existing DCNN without over-fitting. We therefore employed a data augmentation scheme in each image group. Moreover, it is not only the texture of the nodule that indicates the existence of thyroid cancer but also the difference between the textures of the nodule and the neighbouring tissues. Therefore, in our work, unlike the more common augmentation methods such as re-scaling, rotation or contrast-adjusting, we applied a novel approach of augmenting the thyroid samples by cropping samples from the image regions around the centre of the certain thyroid nodule. In each image with the size 1024(±5) × 695(±5), 640(±5) × 440(±5) or 560 × 360 (different between images from different databases), where the region of interest was bounded by 4 pixels with the positions (*x*_*t*_, *y*_*t*_), (*x*_*l*_, *y*_*l*_), (*x*_*b*_, *y*_*b*_) and (*x*_*r*_, *y*_*r*_), nine 256 × 256 sub-images with the labels inherited from the parent image were cropped from the nodule region and the surrounding texture regions. With this method, both the “interesting” tissue and the surrounding tissues are covered in all these samples, making it possible for the DCNN to learn the features from the nodule tissue texture itself and the differences between textures from different tissues. Tables 1 and 2 show the distribution of augmented positive and negative samples in training, validating and testing groups from database 1 and database 2, respectively.

**Table 1:** The distribution of positive and negative cases in training, validating and testing groups of database 1

	Total		Positive		Negative	
	Cases	Samples	Cases	Samples	Cases	Samples
Training	306	2754	256	2304	50	450
Validating	61	549	51	459	10	90
Testing	61	549	50	450	11	99

**Table 2:** The distribution of positive and negative cases in training, validating and testing groups of database 2

	Total		Positive		Negative	
	Cases	Samples	Cases	Samples	Cases	Samples
Training	132	1188	21	189	111	999
Validating	16	144	4	36	12	108
Testing	16	144	4	36	12	108

### Fine-Tuned GoogLeNet Model Generation

Given the database with adequate high-quality samples after pre-processing and augmentation, we employed Deep Learning Caffe library [20] to fine-tune the GoogLeNet to a network that can extract features specifically for thyroid ultrasound images. The initial GoogLeNet convolutional neural network was created by Szegedy et al. [21], using 22 convolutional layers including 9 inception modules and trained on over 1.2 million images from the ImageNet Large Scale Visual Recognition Challenge (ILSVRC) repository [22].

**Fig. 5:**
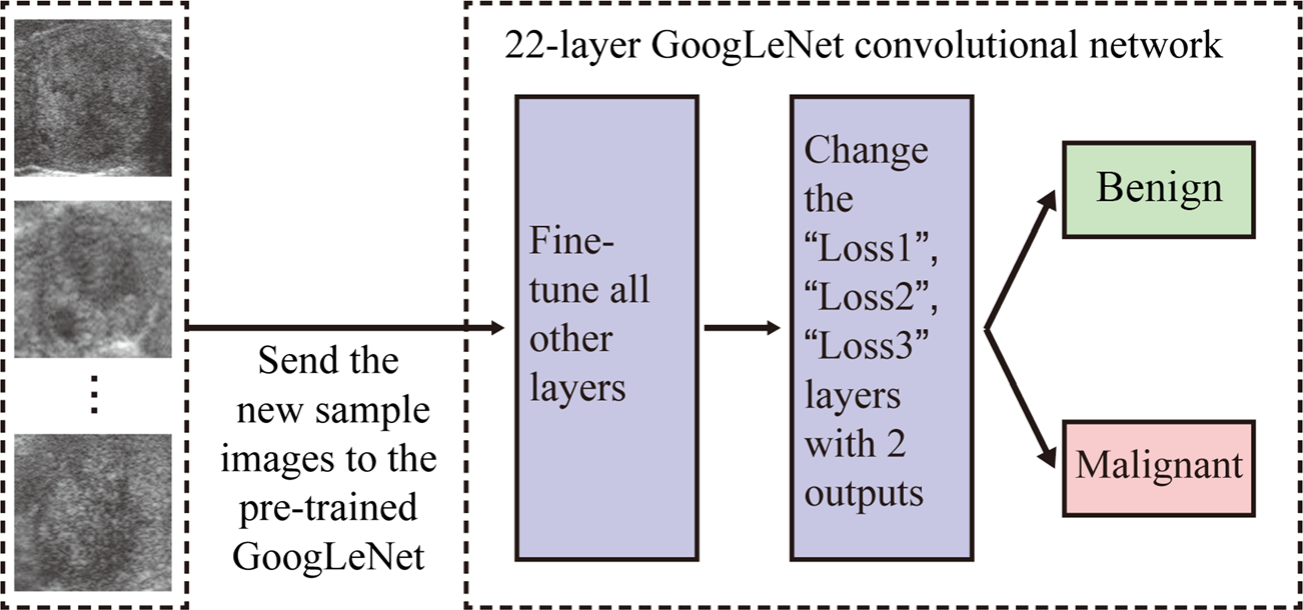
The process of fine-tuning GoogLeNet. The new sample images from the target domain are sent to the pre-trained GoogLeNet, changed the three classification layers and run the GoogLeNet, and then the parameters of the network are fine-tuned automatically

We transferred the parameters of the initial GoogLeNet network by applying them to the training and validating images from our thyroid sonography data. Figure 5 shows the process of the fine-tuning. The “Loss1/classifier”, “Loss2/classifier” and “Loss3/classifier” layers of the DCNN were modified (“num_output” changed from 1000 to 2) to learn only two outputs corresponding to the two classes, i.e. benign or probably malignant. These layers were renamed so that instead of picking the weights from the pre-trained GoogLeNet model, the layers could begin training with random weights. The maximum learning iteration was set to 20,000 as the stopping criterion of the validation testing. Also, the overall learning rate “base_lr” was reduced from 0.01 to 0.001, so that the pre-trained model weights would not distort too quickly and too much. By using training and validating samples from different databases, we generated three different fine-tuned GoogLeNet models.

The first model was generated by fine-tuning the pre-trained GoogLeNet using the training and validating groups from database 1, which contains 2754 samples and 549 samples, respectively.

The second model was generated by fine-tuning the pre-trained GoogLeNet using the training and validating groups from database 2, which contains 1188 samples and 144 samples, respectively.

The third model was generated by fine-tuning the pre-trained GoogLeNet using the training and validating groups fusing samples from both database 1 and database 2, which contains 3942 samples and 693 samples, respectively.

From the three fine-tuned models, we chose a final model based on validation accuracy. We then applied the network to the testing groups of database 1 and database 2 separately.

### Feature Extraction and Classification Model

After fine-tuning the GoogLeNet, the features were first extracted from the image by the fine-tuned network, then the features were fed to a supervised classifier which then classified them as either “benign” or “probably malignant”. “pool5/7x7_s1” layer of the fine-tuned GoogLeNet was used for feature extraction, which extracted 1024 features from each 256 × 256 image. We used a machine learning algorithm called “Cost-sensitive Random Forest” to classify the resulting features, implemented under the Weka 3.8 environment. For different testing groups, we used the same classifier that cascaded 10 Cost-sensitive Random Forest models, each with 100 trees. However, the cost matrices of the classifiers were different between different testing groups, because each cost matrix set the misclassification cost of the benign class over that of malignant class based on the class distribution in each group.

### Model Assessment

The performance of our proposed classification system was evaluated in terms of classification accuracy, sensitivity (also known as true positive rate (TPR)), specificity (also known as true negative rate (TNR)) and area under receiver operating characteristic (ROC) curve (also known as area under curve (AUC)).

## Results

### Model Selection

Fine-tuned with the image samples from both database 1 and database 2 led to higher validation accuracy than the other two fine-tuned GoogLeNet models trained with the image samples only from database 1 or database 2 (98.55 vs 95.73 vs 93.06%). Therefore, we selected the fine-tuned GoogLeNet model trained with image samples from both database 1 and database 2 to extract the deep features for the following classification step.

### Model Assessment

**Fig. 6:**
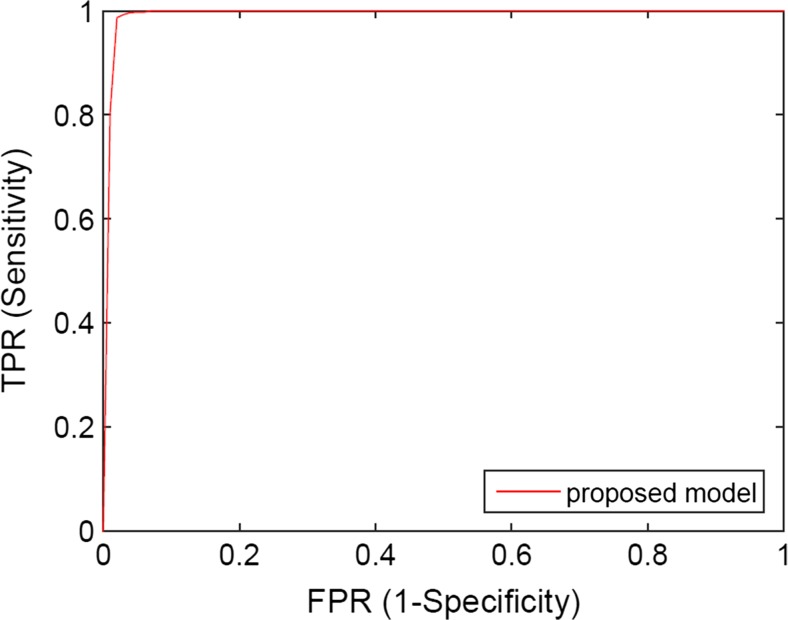
The ROC curves of classifying image samples from database 1

For 10-fold cross-validation, Table 3 shows the classification accuracies, sensitivities and specificities of applying the proposed model to the testing group of database 1, while Fig. 6 shows the corresponding ROC curves. The classification accuracy, sensitivity, specificity and AUC of applying proposed model are 99.13%, 99.70%, 95.80% and 0.9970, respectively.

**Table 3:** The results of classifying testing samples from database 1 by the proposed model (GoogLeNet fine-tuned by samples from both database 1 and database 2)

	Accuracy	Sensitivity	Specificity	AUC
Proposed model	99.13%	99.70%	95.80%	0.9970

**Fig. 7:**
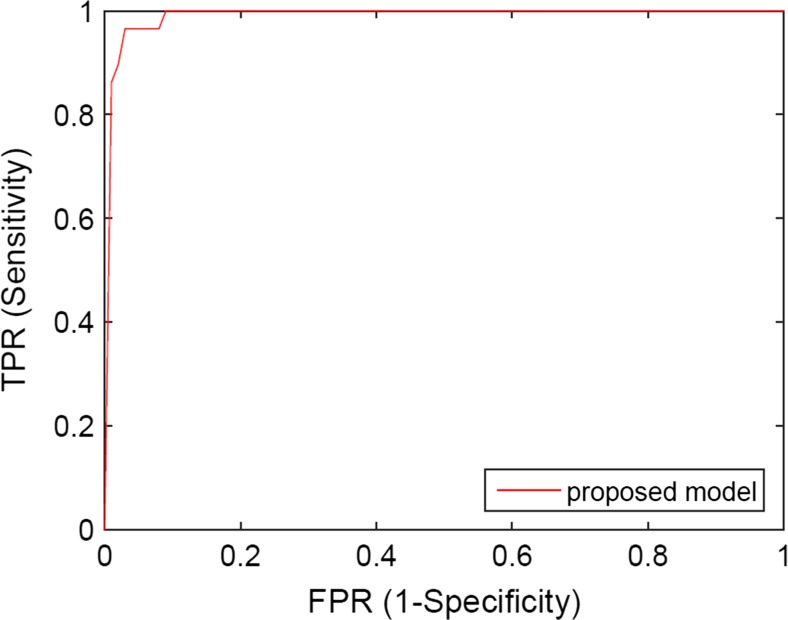
The ROC curves of classifying image samples from database 2

Considering the case of database 2, Table 4 shows the 10-fold cross-validation results of classification accuracies, sensitivities and specificities of applying the proposed model to the testing samples, while Fig. 7 shows the ROC curves. It can be considered that the performance of two models is very similar, where the classification accuracy, sensitivity, specificity and AUC of proposed model are 96.34%, 82.80%, 99.30% and 0.9920.

**Table 4:** The results of classifying testing samples from database 2 by the proposed model (GoogLeNet fine-tuned by samples from both database 1 and database 2)

	Accuracy	Sensitivity	Specificity	AUC
Proposed model	96.34%	82.80%	99.30%	0.9920

## Discussion

From the above experiments, fine-tuning the existing DCNN proves to have the advantage of needing less training samples to generate a domain-specific deep learning network, which can effectively extract high-level features for thyroid ultrasound images and classify malignant and benign thyroid images with high accuracy.

The image pre-processing proved to be effective in improving the fine-tuning of the DCNN: (1) the image normalization can calibrate the details of images from different sources to the same scale, and (2) the removal of the artifacts can recover the textures overlapped by the markers made by the radiologists or doctors, avoiding having the network learn the artifacts. In previous studies, there were millions of training images for the transfer learning of the DCNN. The large quantity of the training images could prevent the artifacts and textures with different scales interrupting the neural network learning; therefore, pre-processing images before transfer learning was not so necessary. However, in our work, we prove that the accurate transfer learning of DCNN can be achieved by using limited but higher quality training images after pre-processing step.

The data augmentation step also proves to be effective in fine-tuning the existing DCNN. Although we got training samples with higher quality showing the features more clearly, the millions of parameters available to be tuned for the network still need thousands of samples to prevent the over-fitting in transfer learning. Prior studies on the impact of database size in transfer learning using DCNN indicated that a suitable size of database for transfer learning could range from 500 to 1000 samples per class [23]; therefore, we augmented the image data to have enough number of training samples. Different from the traditional augmentation methods like re-scaling, rotation or contrast-adjusting, we novelly augmented the thyroid samples by cropping different regions from the image containing both the thyroid nodules and the surrounding tissues, so the network can learn the features from the thyroid nodule itself and the differences between textures from different tissues. Further, we found in our experiments that the distribution of image cases (malignant vs benign) in the training group was imbalanced so the fine-tuned model inadequately reflected the features, so we fused two databases to guarantee that there were enough training samples for each class in the database. The performance of the fine-tuned network is much improved in generally classifying the thyroid images, as we achieved 99.13 and 96.34% classification accuracies for two different testing groups of images from two different databases, respectively.

Our proposed classification system is the novel and exploratory work in applying the deep learning to thyroid nodule assessment problem since it is the first attempt to combine the deep learning method with human assessment-based TI-RADS scoring system. Table 5 compares the performance of several state-of-the-art CAD systems with our proposed model in classifying benign and malignant thyroid nodule images. As mentioned in a recent study [29], specificity of 85.33% and AUC of 0.9135 are achieved by the experienced radiologist, which none of their machine learning models could achieve. Although our work is not directly comparable with these models, we demonstrate that the classification of thyroid ultrasound images by our proposed deep learning-based system could be an efficient method to assess the thyroid nodules, with higher classification accuracy on large size of database.

**Table 5:** Comparison of different CAD system in classifying benign and malignant thyroid nodule images

Method	Feature	Machine learning	Testing samples	Accuracy
Lim et al. [24]	Size, margin, echogenicity, cystic change	Artificial neural network	190 thyroid lesions	93.78%
Savelonas et al. [25]	Shape features	Nearest neighbour (k-NN)	173 longitudinal in vivo images	93.00%
Iakovidis et al. [26]	Fuzzy intensity histogram	SVM	250 thyroid ultrasound images	97.50%
Legakis et al. [27]	Texture features, shape features	SVM	142 longitudinal in vivo images	93.20%
Luo et al. [28]	Strain rate waveform’s power spectrum	Linear discriminant analysis	98 nodule image sequences	87.50%
Acharya et al. [7]	Higher-order spectra features	Fuzzy classifier	80 sample 3D images	99.10%
Proposed model	Deep learning features	Cost-sensitive Random Forest classifier	693 sample images	99.13%

Our study was limited in only classifying the thyroid nodules into two classes of probably malignant and presumed benign. We do not yet have enough samples with all the six different TI-RADS scores to attempt to learn a model that can predict a finer-granularity score. The regions of interest indicating the thyroid nodules were marked by radiologist rather than detected by the computer-aided system automatically, so the performance of transfer learning and the following classification may still highly depend on the experience and knowledge from doctors. Moreover, the proposed system was trained using TI-RADS scores as reference; however, we did not analyse deeply the relationship between TI-RADS scores from radiologists and actual benign/malignant results from pathology.

### System and Running Time

The system we used to fine-tune the GoogLeNet and classify the testing images with the fine-tuned GoogLeNet was MacOS 10.12, with the processor Intel Core i5 at 2.70 GHz, the memory 8192 MB 1600 MHz DDR3 and the CPU only. The running time of fine-tuning GoogLeNet models was 202.27 min with 3942 training samples and 693 validating samples.

## Conclusion

In summary, we propose a thyroid image classification method by fine-tuning an existing deep convolutional neural network GoogLeNet. We demonstrated that two methods can effectively improve the performance of fine-tuning an existing DCNN: (1) pre-processing the image samples to normalize the texture scales and remove artifacts and (2) augmenting the image samples by including both nodule textures and the surrounding textures. Our research work has shown that deep learning, especially fine-tuning an existing deep learning network, can improve the performance of thyroid nodule assessment as a computer-aided diagnosis system.
